# Metabolic Insights From Composition-Based Analysis of Kidney Stones: A Retrospective Study of 506 Patients

**DOI:** 10.7759/cureus.101450

**Published:** 2026-01-13

**Authors:** Sadik Portakal, Basri Cakiroglu, Bekir Sami Uyanık, Selami Aydin, Ali Egemen Avci

**Affiliations:** 1 Family Medicine, Hisar Intercontinental Hospital, Istanbul, TUR; 2 Urology, Üsküdar University Faculty of Medicine, Istanbul, TUR; 3 Urology, Hisar Intercontinental Hospital, Istanbul, TUR; 4 Biochemistry, Hisar Intercontinental Hospital, Istanbul, TUR; 5 Internal Medicine, Üsküdar University Faculty of Medicine, Istanbul, TUR

**Keywords:** calcium oxalate, ftir spectroscopy, metabolic evaluation, nephrolithiasis, stone composition, uric acid

## Abstract

Background: Kidney stone disease is a highly prevalent condition with increasing global incidence and recurrence. The stone composition reflects underlying metabolic and biochemical alterations, and its evaluation may provide clinically relevant information for effective management.

Aim: To evaluate the distribution of stone composition in a large surgical cohort and explore its association with routinely assessed metabolic parameters and recurrence patterns.

Methods: This retrospective single-center study included 506 adult patients who underwent surgical stone removal between 2018 and 2024. The stone composition was determined using Fourier-transform infrared (FTIR) spectroscopy and classified as calcium oxalate monohydrate (COM), calcium oxalate dihydrate (COD), uric acid (UA), or a mixed type. Available serum (calcium, uric acid, glucose) and 24-hour urinary parameters (calcium, oxalate, citrate, uric acid) were evaluated. Subgroup analyses compared first-episode and recurrent stone formers.

Results: A total of 506 patients were included in the final analysis. Calcium oxalate dihydrate (COD) was the most prevalent stone type, accounting for 307 cases (60.7%), followed by calcium oxalate monohydrate (COM) in 117 (23.1%), calcium/magnesium phosphate (CaP) in 46 (9.1%), uric acid stones in 31 (6.1%), and cystine stones in 5 patients (1.0%). The mean age of the cohort was 37.9 ± 11.4 years, and males comprised 73% of the study population. Patients with uric acid stones were significantly older than those with other stone types (p< 0.001), while body mass index did not differ significantly across stone groups (p> 0.05).

Conclusion: Stone composition assessed by FTIR analysis demonstrates meaningful associations with selected metabolic abnormalities and recurrence status. Rather than functioning as a standalone decision tool, composition-guided interpretation combined with comprehensive metabolic evaluation may support more informed patient follow-up and preventive counselling.

## Introduction

Urolithiasis, commonly known as kidney stone disease, has a lifetime prevalence approaching 10-15% in developed regions and shows an increasing trend due to dietary changes, obesity, metabolic syndrome, and sedentary lifestyles [1,2]. Beyond its acute clinical burden, recurrent stone formation contributes to chronic kidney disease, repeated urinary tract infections, and a significant decline in patients’ quality of life [3,4].

Kidney stone formation is a multifactorial process involving genetic, metabolic, environmental, and dietary factors [5,6]. The majority of stones are calcium-based, particularly calcium oxalate (CaOx) stones, followed by uric acid, struvite, and cystine stones [7-9]. Accurate determination of stone composition provides valuable etiological insights and has become a central component of management based on contemporary guidelines [10].

Metabolic evaluation, including serum biochemical tests and 24-hour urine analysis, remains fundamental for both diagnostic assessment and the prevention of recurrence [11]. Common abnormalities, such as hypercalciuria, hypocitraturia, hyperoxaluria, low urine volume, and acidic urine pH, significantly increase the lithogenic risk and inform preventive strategies [12-14]. However, although many existing studies primarily focus on selected patient groups, such as recurrent CaOx stone formers, first-time stone formers, and other clinically relevant stone subtypes, they remain relatively underrepresented [15,16].

Moreover, despite calcium oxalate monohydrate (COM) and calcium oxalate dihydrate (COD) stones sharing a similar chemical composition, there is growing interest in the potential clinical and metabolic differences between these subtypes. The current literature is heterogeneous, and further clarification is needed to determine whether compositional patterns reflect distinct clinical characteristics or secondary indicators of broader metabolic alterations [17,18].

Therefore, this study aimed to evaluate the stone composition using Fourier-transform infrared (FTIR) spectroscopy and investigate its relationship with routinely assessed metabolic parameters in a broad cohort of patients with stone disease. By comparing the compositional profiles across major stone categories and assessing the differences between first-time and recurrent stone formers, we aim to provide practical, clinically relevant insights that may support more informed follow-up and preventive decision-making in patients with kidney stones.

## Materials and methods

Study design and ethical approval

This retrospective study was conducted in accordance with the principles of the Declaration of Helsinki and was approved by the Institutional Ethics Committee of Hisar Intercontinental Hospital (Approval No: 25-32; Date: 16.03.2025). Given the retrospective design, the requirement for obtaining informed consent was waived by the ethics committee, and the manuscript was subsequently made publicly available for discussion on Research Square on September 23, 2025.

Patient selection

Inclusion Criteria

Patients were eligible for inclusion if they fulfilled the following criteria:

Age ≥18 years; diagnosis of urolithiasis (renal, ureteral, or bladder calculi) managed with extracorporeal shock wave lithotripsy (ESWL), percutaneous nephrolithotomy (PCNL), retrograde intrarenal surgery (RIRS), or ureterorenoscopy (URS); stone composition analysis performed using Fourier-transform infrared (FTIR) spectroscopy; availability of metabolic evaluation including 24-hour urine collection and serum biochemical testing; complete demographic, laboratory; follow-up data documenting recurrence status.

Exclusion Criteria

Patients were eligible for exclusion if they fulfilled the following criteria:

Age <18 years and patients with missing or incomplete stone composition or metabolic evaluation data; cases with indeterminate or mixed stone composition without a clearly dominant crystalline component; pregnant patients or individuals with a single-functioning kidney; patients with systemic diseases known to significantly alter calcium or oxalate metabolism (e.g., primary hyperparathyroidism, sarcoidosis, multiple myeloma, renal tubular acidosis); Inadequate 24-hour urine collections, defined by low creatinine excretion (<15 mg/kg/day in men or <10 mg/kg/day in women).

These exclusions were applied to minimize confounding factors and ensure the validity and reproducibility of the metabolic analyses.

Data collection and variables

Clinical, demographic, and laboratory data were retrospectively extracted from electronic medical records at the time of the stone evaluation. Demographic variables included age, sex, body mass index (BMI), family history of urolithiasis, recurrence status, and previous stone-related interventions. Recurrence was defined as at least one previous stone episode confirmed radiologically or surgically.

Serum biochemical parameters, including albumin-corrected calcium, uric acid, and creatinine levels, were obtained from fasting blood samples collected during metabolic evaluation.

Twenty-four-hour urine collections were performed under standard outpatient conditions. The urinary parameters analyzed included total urine volume, calcium, oxalate, citrate, magnesium, phosphate, sodium, potassium, and urinary pH. All laboratory measurements were performed using standardized institutional protocols.

Urine Collection Protocol

The patients maintained their routine diet and daily habits. Urine was collected and stored under refrigeration at 4°C and promptly transported to the central laboratory. Spot urine pH was measured upon arrival, and the mean pH was recorded. All measurements were performed using standardized automated analyzers with rigorous internal and external quality control.

Statistical analysis

Statistical analyses were performed using IBM SPSS Statistics for Windows, Version 26.0 (IBM Corp., Armonk, NY, USA). Data distribution was evaluated using the Kolmogorov-Smirnov test. Normally distributed variables are expressed as mean ± standard deviation (SD) and were compared using one-way analysis of variance (ANOVA) with Tukey’s post hoc test. Non-normally distributed variables are reported as medians (interquartile range, IQR) and compared using the Kruskal-Wallis test with Dunn’s post hoc correction. Categorical variables were analyzed using the chi-square or Fisher’s exact tests, as appropriate. Statistical significance was set at p< 0.05.

## Results

Patient demographics and stone composition

A total of 506 patients met the inclusion criteria and were included in the final analyses. FTIR-based evaluation demonstrated that calcium oxalate dihydrate (COD) was the most common subtype, identified in 307 patients (60.7%), followed by calcium oxalate monohydrate (COM) in 117 (23.1%), calcium/magnesium phosphate (CaP) in 46 (9.1%), uric acid stones in 31 (6.1%), and cystine stones in 5 patients (1.0%). The mean age of the cohort was 37.9 ± 11.4 years, and males accounted for 73% of the population. Patients with uric acid stones were significantly older than those with other stone types (p< 0.001). No significant intergroup differences were observed in the BMI (p> 0.05). The detailed demographic characteristics are presented in Table 1.

**Table 1:** Demographic and Clinical Characteristics by Stone Type (mean ± SD). Age is presented in years and BMI in kg/m².Values are reported as mean ± standard deviation unless otherwise stated. COD: calcium oxalate dihydrate; COM: calcium oxalate monohydrate; CaP: calcium phosphate.

Stone Type	Total (n)	Age (mean ± SD)	BMI (mean ± SD)	Male (n)	Recurrence (n)
COD	307	37.4 ± 11.2	25.8 ± 4.2	237	124
COM	117	35.0 ± 10.0	26.0 ± 4.7	85	58
CaP	46	35.4 ± 11.8	26.1 ± 3.9	33	21
Cystine	5	34.8 ± 9.8	25.9 ± 4.7	3	5
Uric Acid	31	49.2 ± 15.4	26.9 ± 4.7	20	25

Stone recurrence and family history

Overall, 46% of the patients experienced at least one documented recurrence. Recurrence rates differed significantly between stone categories (p< 0.001), being highest among patients with cystine stones (100%) and uric acid stones (81%), whereas recurrence rates ranged between 40-50% among calcium-based stone formers. A family history of urolithiasis was present in 21% of patients, with no statistically significant variation across subgroups (p> 0.05).

Serum Biochemical Profiles

Serum biochemical parameters varied across stone types (Table 2).

**Table 2:** Serum and 24-Hour Urinary Parameters by Stone Type (mean ± SD). Serum units: Calcium (mg/dL), Uric Acid (mg/dL), Creatinine (mg/dL) Urine units: Calcium (g/day), Oxalate (mg/day), Citrate (mg/day), Magnesium (g/day), Phosphate (g/day), pH (no unit) pH: potential of hydrogen; mg/dL: milligrams per deciliter; g/day: grams per day. COD: calcium oxalate dihydrate; COM: calcium oxalate monohydrate; CaP: calcium phosphate.

Stone Type	Serum Ca (mean ± SD)	Serum Uric Acid (mean ± SD)	Serum Creatinine (mean ± SD)	Urine Ca (mean ± SD)	Urine Oxalate (mean ± SD)	Urine Citrate (mean ± SD)	Urine Magnesium (mean ± SD)	Urine Phosphate (mean ± SD)	Urine pH (mean ± SD)
COD	9.41 ± 0.52	5.91 ± 1.33	1.09 ± 3.58	0.34 ± 0.2	24.66 ± 13.71	434.54 ± 276.15	1.17 ± 1.14	0.92 ± 0.32	5.97 ± 0.93
COM	10.2 ± 0.53	5.9 ± 1.46	0.93 ± 0.36	0.36 ± 0.19	25.57 ± 25.62	410.56 ± 221.09	1.13 ± 0.49	0.98 ± 1.26	5.85 ± 1.0
Ca-Mg-pO_4_	8.99 ± 1.54	5.69 ± 1.3	1.05 ± 0.44	0.33 ± 0.2	24.55 ± 11.13	403.12 ± 239.3	1.0 ± 0.47	0.84 ± 0.31	6.8 ± 1.18
CaP	9.34 ± 0.69	5.72 ± 1.64	0.82 ± 0.28	0.37 ± 0.19	23.64 ± 10.73	506.43 ± 329.3	1.2 ± 0.4	0.85 ± 0.28	6.29 ± 0.99
Cystine	9.12 ± 0.64	5.44 ± 1.61	0.91 ± 0.12	0.26 ± 0.09	29.57 ± 14.17	717.6 ± 471.39	1.56 ± 0.43	1.09 ± 0.29	6.0 ± 1.73
Uric Acid	9.29 ± 0.52	8.34 ± 2.37	0.87 ± 0.28	0.32 ± 0.17	27.03 ± 10.62	510.25 ± 316.95	1.2 ± 0.47	0.88 ± 0.36	5.18 ± 0.66

Serum biochemical parameters demonstrated distinct patterns according to the stone composition. Patients with calcium oxalate monohydrate (COM) stones had significantly higher serum calcium levels than those in all other stone groups (p< 0.001). As expected, individuals with uric acid stones exhibited markedly elevated serum uric acid levels (p< 0.001). In contrast, serum creatinine levels did not differ significantly among the different stone subtypes (p=0.457).

**Figure 1:**
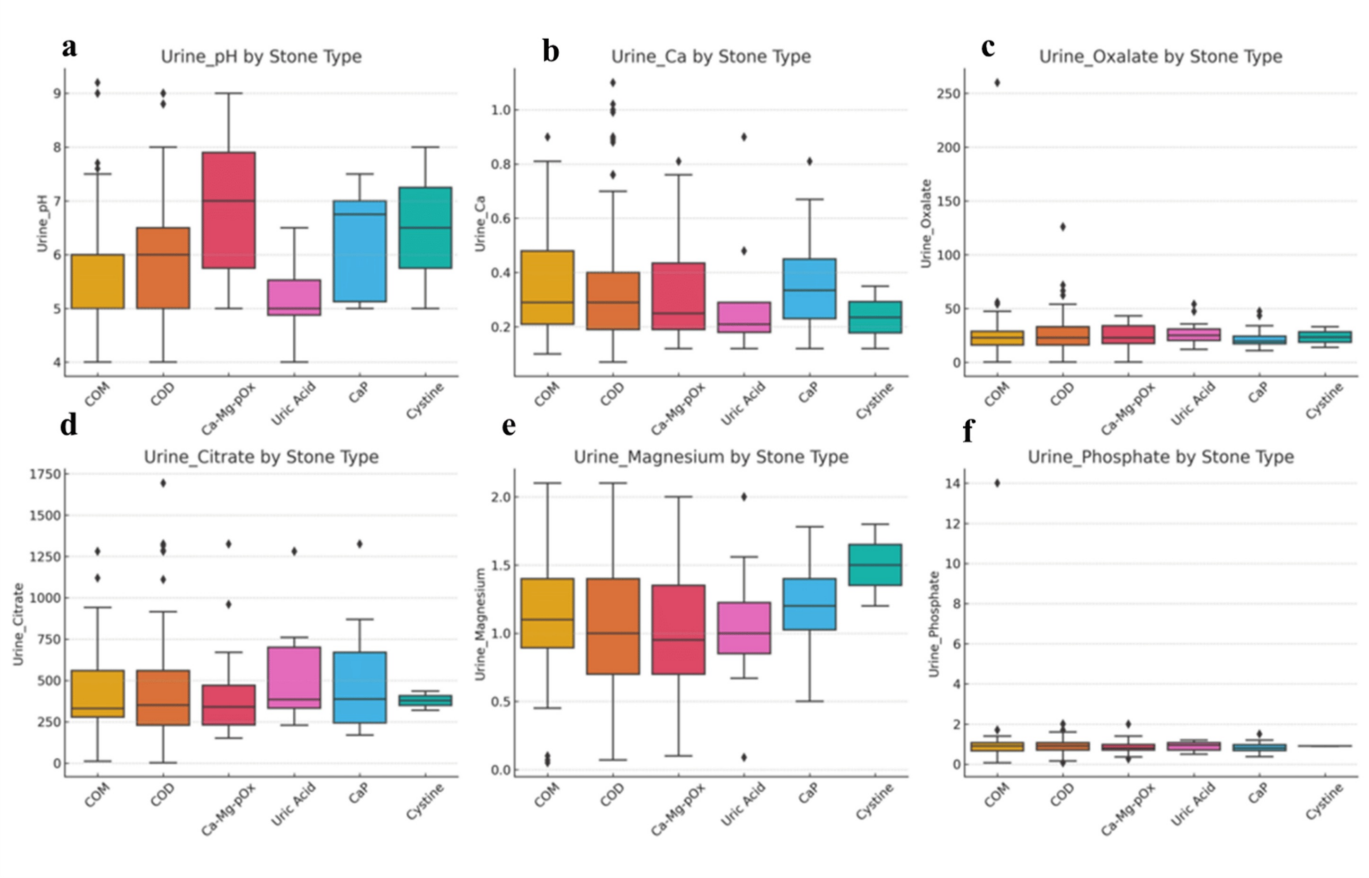
Boxplots of Urinary Parameters by Stone Type. (a) Urinary pH distribution according to stone type, demonstrating lower pH values in uric acid stones and relatively higher pH values in calcium phosphate and cystine stones. (b) Urinary calcium (Ca) levels by stone type, with higher median values observed in calcium-containing stones than in uric acid and cystine stones. (c) Urinary oxalate excretion according to stone composition, showing increased variability, particularly in calcium oxalate stone formers. (d) Urinary citrate levels by stone type, highlighting lower citrate excretion in calcium oxalate stones and relatively higher values in uric acid stone formers. (e) Urinary magnesium distribution across stone types, with higher levels observed in cystine and calcium phosphate stones. (f) Urinary phosphate levels according to stone type, demonstrating relatively higher phosphate concentrations in calcium phosphate stones. COM: calcium oxalate monohydrate; COD: calcium oxalate dihydrate; Ca–Mg–PO₄: calcium/magnesium phosphate stones; Uric Acid: uric acid stones; CaP: calcium phosphate stones; Cystine: cystine stones.

Analysis of 24-hour urine profiles revealed composition-related differences (Figure 1). Urinary pH varied significantly across stone groups (p< 0.001), with the lowest mean pH observed in uric acid stone formers (5.2) and the highest in calcium phosphate (CaP) stone formers (6.6). Urinary calcium and oxalate excretion were comparable among the stone types (p=0.626 and p=0.998, respectively). Although urinary citrate excretion tended to be lower in patients with COM stones, this difference was not statistically significant (p=0.064). Similarly, the urinary magnesium and phosphate levels were comparable across all groups (p> 0.05).

Metabolic abnormalities

The prevalence of metabolic abnormalities is presented in Table 3. The key observations include: Low urine volume (<2000 mL/day) was frequent overall (~70%) and most common in patients with uric acid stones (77.4%). Hypocitraturia (<320 mg/day) was most frequently observed in patients with COM stones (60.7% of patients). Hypercalciuria (>300 mg/day) was present in 42% of the cohort and predominantly detected among CaOx stone formers. Hyperoxaluria (>45 mg/day) was relatively uncommon, occurring in 6% of patients.

**Table 3:** Prevalence of Metabolic Abnormalities. COM: calcium oxalate monohydrate; COD: calcium oxalate dihydrate; CaP: calcium phosphate.

Metabolic Abnormality	COM	COD	CaP	Uric Acid	Cystine
Low urine volume (<2000 mL/day), n (%)	82 (70.1%)	213 (69.4%)	33 (71.7%)	24 (77.4%)	3 (60.0%)
Hypocitraturia (<320 mg/day), n (%)	71 (60.7%)	161 (52.4%)	21 (45.7%)	14 (45.2%)	1 (20.0%)
Hypercalciuria (>300 mg/day), n (%)	52 (44.4%)	132 (43.0%)	19 (41.3%)	8 (25.8%)	2 (40.0%)
Hyperoxaluria (>45 mg/day), n (%)	9 (7.7%)	18 (5.9%)	2 (4.3%)	1 (3.2%)	0 (0%)

Summary of Main Findings

The stone composition was significantly associated with age, urinary pH, and selected serum biochemical parameters. Low urine volume and hypocitraturia were the most frequent metabolic abnormalities observed in all stone groups, and recurrence was particularly frequent in cystine and uric acid stone formers than in calcium-based stone formers.

## Discussion

This study provides a comprehensive evaluation of patients with nephrolithiasis by integrating FTIR-based stone composition analysis with serum and 24-hour urine biochemical assessment in a large real-world cohort. A particular strength of this study is the detailed comparison of calcium oxalate subtypes in relation to recurrence status and metabolic abnormalities, offering clinically relevant insights into stone-forming patterns encountered in routine practice.

Metabolic abnormalities in first-time and recurrent stone formers

A prominent finding of this study was the high prevalence of metabolic abnormalities, even among patients with first-time stone formation. As shown in Table 2, abnormalities such as low urine volume, hypocitraturia, and hypercalciuria were frequently observed at the initial presentation. Traditionally, metabolic evaluation has been emphasized mainly for recurrent disease; however, our findings support the earlier observations by Daudon et al. and Moe et al., who reported measurable lithogenic risk factors even after the first stone episode [15,16]. In line with current European Association of Urology (EAU) recommendations, these results reinforce the clinical value of metabolic investigations following the initial stone event [10].

Among the urinary parameters, citrate excretion was lower in patients with recurrent stone disease (Table 2), supporting its inhibitory role in calcium oxalate crystallization [13,14]. Although the mean urinary magnesium levels did not differ significantly between the groups, the lower absolute values observed in recurrent cases may correspond to reduced inhibitory capacity, as suggested in previous studies [19,20]. Low urine volume was highly prevalent across all groups (Figure 1), reaffirming that adequate hydration is a fundamental, preventive measure.

COM and COD Stones

The differences between calcium oxalate monohydrate (COM) and dihydrate (COD) stone formers were mainly reflected in the selected serum and urinary parameters (Table 2). However, most intergroup differences were modest, suggesting overlapping metabolic profiles rather than distinct and fixed biochemical phenotypes.

Beyond Calcium Oxalate; Uric Acid and CaP Stones

These findings support urine alkalinization and dietary purine restriction as key preventive strategies. Conversely, calcium phosphate (CaP) stone formers demonstrated the highest urinary pH values (Figure 1), reflecting an alkaline environment conducive to phosphate crystallization. In patients with CaP stones or persistent metabolic abnormalities, consideration of normocalcemic primary hyperparathyroidism may be appropriate, as emerging evidence suggests clinically relevant metabolic effects despite normal serum calcium levels [21]. Patients with uric acid stones were significantly older and exhibited lower urinary pH and higher serum uric acid levels (Tables 1, 2), consistent with prior reports [22].

Oxalate, Supersaturation, and Recurrence

Although the mean urinary oxalate values were generally within the reference limits (Table 2), even minor elevations may significantly increase supersaturation, particularly in the presence of low urine volume or hypocitraturia (Figure 1) [22,23]. This observation reinforces the multifactorial nature of stone recurrence and highlights the importance of comprehensive metabolic evaluation rather than relying on single parameters.

Clinical implications

As summarized in Tables 1, 2, metabolic abnormalities were common even after the first stone episode. Hypocitraturia and low urine volume were identified as key modifiable risk factors (Figure 1). While stone composition provides valuable diagnostic information, these findings underscore that it should not be interpreted in isolation but integrated with metabolic evaluation to guide individualized prevention and follow-up strategies.

Comparison with previous literature

Variability in COM and COD predominance has been reported previously, and the distribution observed in our cohort (Table 1) aligns with studies suggesting regional influences on calcium oxalate morphology [17,24]. Consistent with the prior literature, uric acid stone formers in our study were associated with older age, metabolic features, and acidic urinary pH (Tables 1, 2) [22]. By integrating FTIR-based stone analysis with structured metabolic data from a large cohort, this study adds to the growing body of evidence supporting the clinical relevance of stone composition when interpreted in conjunction with biochemical profiles.

Limitations and future directions

The main limitations of this study include its retrospective single-center design, lack of dietary standardization during 24-hour urine collection, and incomplete hormonal evaluation (e.g., parathyroid hormone and vitamin D levels). Rare stone types, such as cystine stones, were also underrepresented. Future prospective multicenter studies incorporating dietary assessments, supersaturation indices, and expanded biochemical markers are warranted to refine the recurrence prediction models.

## Conclusions

In this large retrospective cohort study, stone composition emerged not merely as a descriptive characteristic but as a clinically meaningful determinant of biochemical phenotype. Distinct metabolic patterns were observed across stone subtypes: uric acid stones tended to cluster with acidic urine and hyperuricemia, whereas calcium-based stones demonstrated broader metabolic heterogeneity, including low urinary volume and hypocitraturia, even at the first presentation. These findings indicate that metabolic abnormalities are under-recognized and may occur much earlier in the disease course than previously assumed.

Integrating FTIR-based stone analysis with targeted metabolic evaluation provides actionable diagnostic value that extends beyond routine assessments. This combined approach may refine risk stratification, facilitate individualized preventive counselling, and support earlier therapeutic interventions prior to recurrence. Taken together, these data strengthen the rationale for shifting nephrolithiasis management from a reactive, recurrence-driven paradigm to a proactive, etiology-based strategy.
